# Tolerance Mechanisms of Olive Tree (*Olea europaea*) under Saline Conditions

**DOI:** 10.3390/plants13152094

**Published:** 2024-07-29

**Authors:** Mohamed El Yamani, María del Pilar Cordovilla

**Affiliations:** 1 Laboratory of Applied Sciences for the Environment and Sustainable Development, Essaouira School of Technology, Cadi Ayyad University, B.P. 383, Essaouira 40000, Morocco; 2 Center for Advances Studies in Olive Grove and Olive Oils, Faculty of Experimental Science, University of Jaén, Paraje Las Lagunillas, E-23071 Jaén, Spain

**Keywords:** salt stress, tolerance, morphological adaptation, photosynthesis, antioxidant defense, osmotic adjustment, proteins, molecular mechanisms

## Abstract

The olive tree (*Olea europaea* L.) is an evergreen tree that occupies 19% of the woody crop area and is cultivated in 67 countries on five continents. The largest olive production region is concentrated in the Mediterranean basin, where the olive tree has had an enormous economic, cultural, and environmental impact since the 7th century BC. In the Mediterranean region, salinity stands out as one of the main abiotic stress factors significantly affecting agricultural production. Moreover, climate change is expected to lead to increased salinization in this region, threatening olive productivity. Salt stress causes combined damage by osmotic stress and ionic toxicity, restricting olive growth and interfering with multiple metabolic processes. A large variability in salinity tolerance among olive cultivars has been described. This paper aims to synthesize information from the published literature on olive adaptations to salt stress and its importance in salinity tolerance. The morphological, physiological, biochemical, and molecular mechanisms of olive tolerance to salt stress are reviewed.

## 1. Introduction

### 1.1. Importance of the Olive Tree

The cultivated olive tree *Olea europaea* subsp. *europaea* var. *europaea* L. originated from *Olea europaea* subsp. *europaea* var. *sylvestris* (Mill) Lehr by artificial selection from wild populations [1]. This evergreen tree is believed to have been one of the first major domesticated fruit crops in the Old World. In the Chalcolithic Levant, evidence for the existence of the olive tree has been found from about 6800–6300 cal BP [2]. The olive tree originated on the eastern shores of the Mediterranean in what is now southern Turkey, Syria, Lebanon, Palestine, and Israel. From there, it spread to populate all countries bordering the Mediterranean [3]. Since then, it has exerted a notable influence on the Mediterranean economy, history, culture, and environment [4,5,6,7], where the olive tree is considered to be the dominant fruit tree in the area [8].

**Figure 1:**
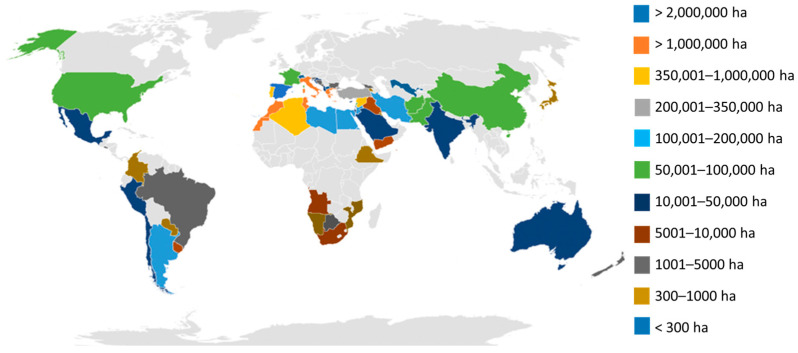
Distribution of the olive groves in the world [7].

Currently, olive cultivation is present in sixty-seven countries across five continents (Figure 1), while its consumption extends to 179 countries. Worldwide, about 11,594,987 ha of olive trees have been documented [9]. Of the olives produced, 13.39% are used for table olives, and 86.61% for olive oil production. Europe has the world’s largest area under olive cultivation and production. However, productivity is highest in Oceania for table olives and olive oil, followed by America for table olives and Europe for olive oil [9] (Suplementary Figure S1).

The olive tree is a species adapted to the semiarid regions of the Mediterranean climate, where it has traditionally been grown in rainfed conditions [10] with a low-density planting system (between 70 and 120 trees/ha) [11]. However, the growing demand for olive products has led to an intensive (200–500 trees/ha) or super-intensive (2500 trees/ha) cultivation model, along with the introduction of irrigation. In 2021, 60% of olive growing production was rainfed, while 40% was irrigated [9] (Supplementary Figure S2).

**Figure 2:**
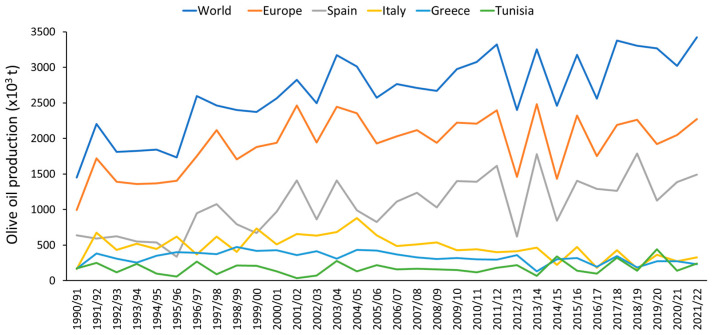
Olive oil production (×10^3^ t) by growing season. Prepared by the authors based on data from the IOC [9].

The Mediterranean region accounts for the majority of olive production, with 97% of the world’s olive oil produced there [12]. Among the top 10 producers of olive oil and table olives, Spain stands out for its high production, accounting for 43.58% of olive oil production and 28.86% of table olive production (Supplementary Figure S3). For olive oil production, Italy (9.61%), Tunisia (7.94%), and Greece (6.77%) also stand out, and for table olive production, Turkey (20.32%), Egypt (19.80%), Algeria (11.06%), and Morocco (5.68%) [13,14]. World olive oil production has doubled in the last three decades (Figure 2). This is due to the balanced composition of the oil and its link with greater longevity and lower cardiovascular disease rate, cancer, and cognitive decline with age [15,16,17]. 

More than 2600 olive cultivars are cultivated worldwide, many of which are traditional and ancient, with little representation in modern olive growing (Table 1). These cultivars have different genetic characteristics and respond differently to biotic and abiotic stresses [18], which offers a wide field of study in the adaptation of an olive grove to a changing environment. 

### 1.2. Problems of Salinity in Relation to Olive Cultivation

Soil salinity affects approximately 900 million ha of cultivated land worldwide [19] (Table 2). Ludwig et al. [20] project that 30% of arable land will be lost in the next 25 years, increasing to 50% by the middle of the 21st century. In saline soils, the accumulation of soluble salts retains water and accentuates the problem of desertification. As a consequence, the electrical conductivity (EC) of the soil increases. A soil is considered saline when it has an EC ≥ 4 dS/m (about 40 mM NaCl), and the percentage of exchangeable sodium is less than 15% [21]. However, the type of plant, the soil–water regime, and climatology influence the threshold value above which harmful effects on the plant occur [22].

In arid and semi-arid climates, high evaporation and insufficient leaching lead to salt accumulation, resulting in the development of saline soils [26]. Additionally, saline groundwater rising by capillary action through the soil profile and subsequent evaporation also contribute to salt accumulation in the soil [27]. Therefore, salinity is one of the main abiotic stresses that severely affect agricultural production in arid and semi-arid regions such as the Mediterranean basin [28,29].

Groundwater contamination is a serious global problem, especially in arid and semi-arid areas. Groundwater salinization can be due to factors such as climate, geology, wastewater recycling, irrigation return flows, septic tank leachates, and saline water intrusion [19,30]. This issue is critical because groundwater is the natural resource that most supports the socio-economic development of communities by providing water for agricultural, domestic, and industrial activities [31]. 

Advancements in irrigated agriculture are indispensable to feed a world population that is likely to exceed 10 billion by 2100 [32]. Olive cultivation has traditionally been rainfed; however, irrigation is becoming increasingly important in the new olive production scenarios. This poses a challenge for the Mediterranean olive basin, where water scarcity limits the availability of freshwater for agriculture, leading to excessive use of saline groundwater and synthetic fertilizers [29,33]. In coastal areas, the use of saline water (EC > 2.0 dS/m) for crop irrigation may be a possible alternative to cope with water scarcity [34]. Using wastewater could also be an interesting practice in sustainable agriculture, as it supplies nutrients that improve soil fertility. However, it can also cause serious salinity issues [35,36,37,38]. Furthermore, according to the Fifth Report of the UN Intergovernmental Panel on Climate Change, precipitation is likely to decrease, increasing the risk of summer drought [39]. This, together with rising temperatures, will exacerbate salinization in the Mediterranean area.

Soil salinization causes salt stress in most cultivated plants, which has a direct impact on food quality and quantity [19]. Salt-tolerant crop cultivars have been developed using transgenic technologies and conventional breeding approaches. However, these two approaches are insufficient, labor- and time-intensive, and alternative technologies need to be used simultaneously to promote sustainable agriculture. In recent years, several strategies have been developed to mitigate the impact of salt stress on crops [40,41,42,43]. 

Saline water intrusion could be mitigated using sustainable strategies such as natural barriers, restoration and conservation of wetlands in inland delta regions, adoption of buffer zones in canals and ditches, and incorporation of aquaculture [43]. Depending on regional characteristics, mangroves, salt marshes, or seagrass meadows may serve as natural barriers [44,45,46]. Wetlands and natural freshwater reservoirs release water to groundwater systems and aquifers, preventing saline water intrusion [47,48]. Buffer zones planted with salt-tolerant vegetation adapted to the local climate help stabilize soil and prevent erosion [49]. Finally, it should be noted that in some crops, such as rice, a rice–shrimp rotation system has been established, which has made it possible to counteract the economic losses due to a decrease in rice crop production as a result of salinity [50,51,52]. 

Soil salinization requires the adoption of sustainable water management practices, such as the promotion of efficient irrigation for crop production (e.g., sub-irrigation and drip irrigation) and rainwater harvesting [43]. In areas with water scarcity and irregular rainfall, water use efficiency can be improved by combining groundwater and surface water resources for agricultural use [13,53], allowing the development of irrigated agriculture and controlling soil salinization problems and shallow water tables [54,55]. Furthermore, the development of irrigated agriculture demands adequate drainage systems to prevent the accumulation of salts in the soil [14,56]. 

The integration of halophytes into the cropping system offers an alternative for salinity management in agricultural soils [57,58]. The use of halophytes as cover crops could improve soil quality and decrease salt accumulation [43]. In addition, the Mediterranean area has a high biodiversity of endemic halophytic plants, providing an alternative group of potential new agricultural products to be cultivated in olive groves [59,60,61].

To mitigate or reduce the impact of salt stress on plants, organic amendments such as biochar, plant growth-promoting rhizobacteria (PGPR), vermicompost, vermi-wash, and biofertilizers have recently been used [42,62,63,64]. PGPBs are found in the root zone of plants in saline soils and promote salt stress tolerance through various mechanisms. These include biofilm formation, production of extracellular polymeric substances, production of phytohormones, and nitrogen fixation, as well as enhancement photosynthesis, photosynthetic pigments, nutrient uptake, and ionic homeostasis in the plant, and increased antioxidant activities under salt stress conditions [65,66,67]. PGPBs promote plant growth by producing hormones such as auxin, cytokinin, and gibberellin and the reduction of ethylene by 1-aminociclopropano-1-carboxilato (ACC) deaminase [65,68,69]. In this line, it has been reported that exogenous application of gibberellic acid, indole-3-acetic acid, salicylic acid, and brassinosteroids can reduce the effect of salinity in the olive tree, improving plant growth, chlorophyll, K^+^, and proline concentration, and/or decreasing Na^+^ and Cl^−^ accumulation in the plant [70,71,72,73,74]. Melatonin is an indolamine that has a similar action to indole-3-acetic acid. Melatonin has been reported to improve plant resistance to salt stress by scavenging reactive oxygen species and enhancing antioxidant enzyme activity, photosynthetic efficiency, and metabolite content, as well as regulating stress-associated transcription factors [75,76,77,78,79]. Other studies have shown that the application of nanoparticles such as ZnO-NP can reduce the harmful effects of salt stress by interacting with transcription factors, inducing the production of certain phytohormones and osmoprotective solutes [80,81,82,83,84].

### 1.3. Overview of Olive Tolerance to Salt Stress

Salt stress tolerance is a multigenic trait involving various mechanisms [85,86]. Olive is generally considered moderately tolerant to salinity [87], although differences in response to salt stress have been observed between cultivars [88,89,90,91,92,93,94].

The mechanisms of salt tolerance in the olive tree are attributable to its ability to limit the transport of salt ions to sensitive shoot organs, causing accumulation in the root, as well as to its capacity to maintain an adequate K^+^/Na^+^ ratio in actively growing tissues [17,89,90,95,96,97]. In addition, olive trees can improve their tolerance to salt stress through osmotic adjustment and reactive oxygen species (ROS) scavenging at the cellular level [58,85,93,94,98,99,100,101].

Sodium ions enter the root cells passively via non-selective ion channels and K^+^ transporters [102,103,104]. In plants under salt stress, Na^+^ accumulation in tissues reduces the membrane potential and consequently facilitates Cl^−^ uptake under a chemical gradient [105]. Excess Na^+^ is transported from the root to the shoot by transpiration, damaging the growing tissues [106]. For plant survival, it is essential to maintain Na^+^ homeostasis under salt stress. The ions exclusion mechanism in leaves has been described in other woody plants and is closely related to the ability to withstand water deficit [107]. The most sensitive olive cultivars to salt stress have less capacity for Na^+^ exclusion [34,108,109,110,111,112]. This tolerance mechanism is shown by most olive cultivars at levels of 50 mM NaCl or lower [90,112]. Studies on the cultivar ‘Leccino’ at 60 mM NaCl indicated that Na^+^ uptake by the root starts within a few hours of salt treatment; however, Na^+^ transport to the shoot requires more time [113,114].

The accumulation of Na^+^ in the vacuole stimulates the synthesis of osmoprotective solutes to balance the osmotic potential between the cytoplasm and the vacuole [115,116,117,118]. In olive trees, the accumulation of osmolytes in the cytoplasm is an adaptive mechanism against salt stress, helping to regulate osmotic potential from the cellular level to the whole plant [57,85,92,119,120,121,122]. Additionally, salinity induces oxidative stress by generating ROS that can damage proteins, DNA, and membrane lipids [123]. Several studies have addressed the oxidative stress caused by salinity in olive trees [57,91,92,124].

**Figure 3:**
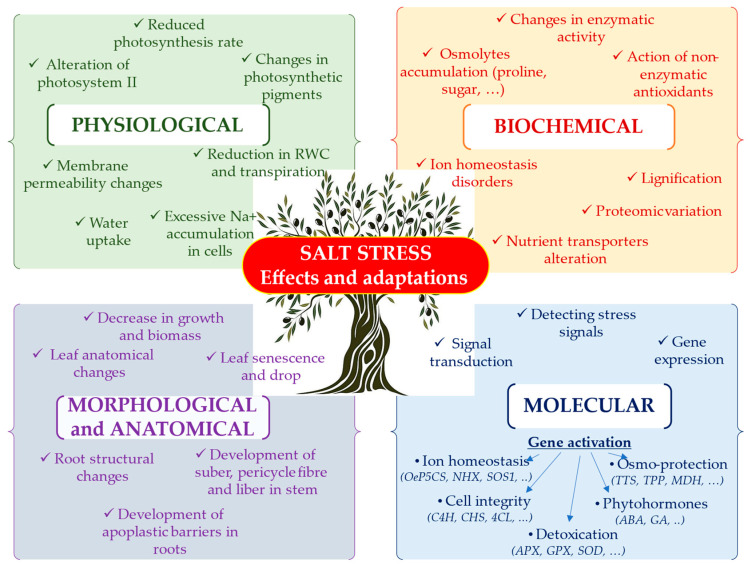
An overview of morphological, anatomical, physiological, biochemical, and molecular mechanisms of salt stress tolerance in olive trees.

Given predictions that soil salinity could increase in the Mediterranean basin, where the olive tree is an important industrial crop, and considering the serious social and economic consequences of a decrease in olive production, this review aims to provide an overview of advances in research on salt stress in the olive tree, promoting the sustainable development of the crop. Indeed, the olive tree deploys a panoply of mechanisms to ensure its survival, resilience, and productivity in saline environments. These mechanisms include morphological, anatomical, physiological, biochemical, and molecular adaptations (Figure 3).

## 2. Morphological, Anatomical, and Physiological Adaptation of the Olive Tree to Salt Stress

### 2.1. Morphological and Anatomical Adaptation

**Figure 4:**
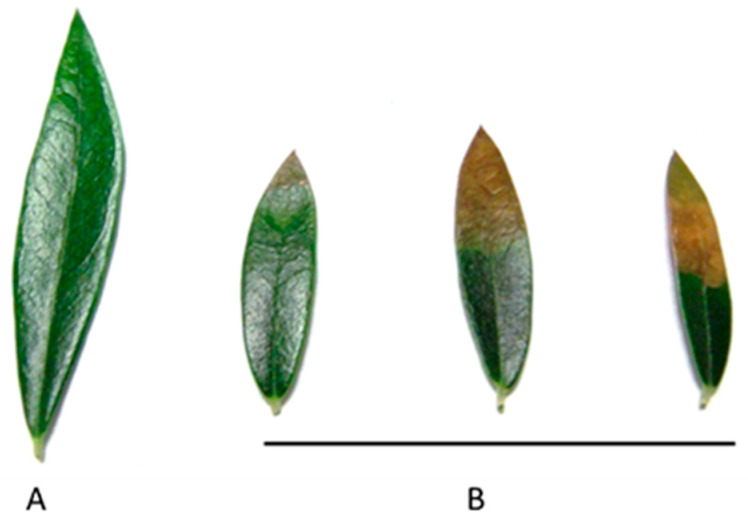
(**A**) Control leaf and (**B**) leaves with ion accumulation. Necrosis starts at the leaf apex and progresses towards the base [127].

Olive trees can undergo morphological and anatomical changes in response to salinity. Plant growth is an often-used morphological indicator in the evaluation of salt stress, although the degree of biomass decline depends on the species and cultivar [90,120,123,124]. In olive plants, growth can be decreased with 50 mM NaCl [57,89,96,98,123,124,125,126]. This decrease in growth due to salinity affects all plant organs [58,91,97,98,124]. Studies have shown the effect of salt is more prominent in the aerial part (shoot + leaves) than in the root at NaCl concentrations above 50 mM, resulting in a higher root/shoot ratio with increasing salinity. Senescent leaf drop may contribute to this effect [89,90,96,98]. Furthermore, the elongation of root cells and shoots exhibits varying tolerance to salt stress [79,127]. The accumulation of salt ions in leaves is manifested by apical burns, necrosis, and old leaf drop [57,91,128,129,130,131,132,133,134] (Figure 4). These toxicity symptoms usually appear at an advanced stage of leaf salinity damage [89,135,136].

The exclusion of saline ions from the shoots of olive trees is related to structural changes in roots. In studies with ‘Barnea’ olive trees, which is considered a moderately salinity tolerant cultivar, an effective exclusion mechanism is the high turnover rate of the Na^+^ hyperaccumulating fine roots [100]. In higher root orders, salt accumulation and mortality are lower, although they decrease in length and increase in diameter [99,100]. The increase in diameter is due to an increase in the size of the periderm cells, which accumulate Na^+^ inside them and act as an apoplastic barrier against ion advance. In this way, the exclusion of salt ions from vascular tissues is enhanced [100,113]. In addition, these roots decrease the stele area with salinity and increase the diameter of the xylem vessels, increasing hydraulic conductivity in the root [100,137,138,139]. Consequently, olive tree resistance to salinity has a structural basis.

The higher ion concentration in the outer root layers has also been documented for the primary roots of young olive trees grown under salt stress. In these primary roots, root diameter and cortical cell density decrease, resulting in reduced salt ion uptake. Moreover, apoplastic barriers develop in the exodermis and endodermis of sensitive (‘Leccino’) and tolerant (‘Frantoio’) cultivars by accumulation in suberin lamellae [113,140,141]. Rossi et al. [113] suggest that the speed of apoplastic barrier development could be related to the reduced absorption and translocation of large amounts of Na^+^ into the shoot. In the ‘Leccino’ cultivar, early translocation of Na^+^ to the aerial part has been observed. This may be due to the decreased expression of ATPase 1, SOS1 (plasma membrane Na^+^/H^+^ antiporter), vacuolar V-type ATPase, and NHX (vacuolar Na^+^/H^+^ antiporter) genes in the early phase of root salt response [114].

Olive leaves also show anatomical modifications in response to salinity. Leaf and upper epidermis thickness may increase under salt stress [126,142,143,144]. As leaf thickness increases under salinity stress, internal surface area per unit leaf area increases, internal resistance decreases, and CO_2_ absorption and water retention potential remain at higher levels [126]. The number of trichomes, the development of collenchyma and sclereids, and the number of conducting vessels increased with salinization [145,146]. An increase in leaf palisade parenchyma under salt stress has been described in different olive cultivars (‘Chondrolia Chalkidikis’, ‘Manzanilla de Sevilla’ and ‘Kalamon, Meski’) [142,146]. However, Fayed et al. [145], in studies with ‘Picual’, ‘Kalamata’, ‘Hamed’ and ‘Toffahi’ cultivars, reported a reduction in the palisade parenchyma and midrib thicknesses. In stems, suber, pericycle fiber, and liber development were observed under salt stress [146].

### 2.2. Photosynthesis Response

The slower growth of glycophytes under salt stress results from two successive processes: osmotic stress and ionic toxicity in the plant tissue. First, water uptake by the plant is reduced, followed by the entry of excess soil ions into the plant, damaging plant cells. Salt ions inhibit photosynthesis, alter ionic homeostasis, and cause peroxidation of lipid membranes [85,147,148,149,150]. 

Olive trees under salinity conditions are subject to important physiological changes, such as a marked decrease in the rate of photosynthetic assimilation (A), stomatal conductance (gs), and transpiration (E) [57,89,117,144,150,151,152,153,154,155]. Reduced growth under salinity stress is associated with reduced photosynthesis [89,91,126,154]. Studies with different cultivars have shown that the photosynthetic rate decreased with an increasing level of salt stress [17,57,89,90,91,94,97,110,144,156,157,158]. The effect of salinity on the CO_2_ assimilation rate varies with salt concentration, cultivar, duration of stress, and plant age [126]. For example, the photosynthetic rate decreased in 4-month-old plantlets of the cultivar ‘Frantoio’ with 12.5 mM NaCl for 35 days [109]. However, in 1-year-old plants of the cultivars ‘Koroneiki’, ‘Kalamata’, ‘Amphisis’ and ‘Kothreiki’ it did not decrease with 50 mM NaCl for 5 months [89]. Similar results were described for 1-year-old plants of the ‘Chemlali’ cultivar grown with 50 mM NaCl for 2 months [146]. Most cultivars studied show a decrease in CO_2_ assimilation with 100 mM NaCl [57,89,91,109,144,155,159,160,161]. Greater inhibition of the CO_2_ assimilation rate has been reported in olive cultivars with high rates of photosynthesis and stomatal conductance [94,144,161,162]. In several studies, it has been observed that photosynthesis inhibition is lower in the more tolerant cultivars compared with the more sensitive ones [94,144]. Furthermore, in the salt-tolerant cultivar ‘Chemlali’, a more rapid activation of the stress response was displayed than in the moderately tolerant ‘Koroneiki’, resulting in a later inhibition of photosynthesis [94].

The reduction in the osmotic potential of the olive leaf under salt stress leads to stomata closure [94,109]. This may be due to the accumulation of Na^+^ ions in stomatal guard cells and can lead to a decrease in internal CO_2_ availability [57,126,144]. Consequently, the decline in photosynthesis under salt stress may be caused by a decrease in gs due to hydroactive stomatal closure [57,94,153,155,157]. This limitation of CO_2_ assimilation by stress can result in the production of ROS by over-reduction of the photosynthetic electron transport chain [91,154,157,163]. However, when leaf turgor potential affects stomatal conductance and net CO_2_ assimilation rate to different degrees, the ionic (as distinct from the osmotic) component of salt stress would control gas exchange performance [159]. In the cultivars ‘Arbequina’, ‘Royal de Cazorla’, ‘Koroneiki’, ‘Fadak86’, ‘Frantoio’, and ‘Leccino’, photosynthesis and stomatal conductance decreased, while the internal CO_2_ concentration increased under salt stress [91,109,154,159]. In this case, reduced photosynthesis due to salinity could be attributable to alterations in photosynthetic metabolism and the inhibition of Calvin cycle enzymes [91,148,154,164,165,166].

The maximum efficiency of photosystem II photochemistry (Fv/Fm) can decrease up to 0.5 in olive plants under salt stress [144,162]. However, in the early phase of salt stress with 60 mM NaCl (7 days of treatment), no decrease in Fv/Fm of the olive cultivar ‘Leccino’ has been observed [114]. In the ‘Gemlik’ and ‘Kilis’ cultivars, Fv/Fm was unaffected after 15 days of growth with 100 mM NaCl but decreased by about 6% with 200 mM NaCl. In contrast, in the ‘Ayvalik’ cultivar, Fv/Fm decreased by 7% after 15 days with 100 mM NaCl [92] and by 17% in ‘Chétoui’ after 21 days with 200 mM NaCl [117]. Similar results were described for ‘Frantoio’ (35 days at 200 mM NaCl) [162]. Under long-term salt stress conditions, the Fv/Fm ratio decreased only at high salt levels (200 mM NaCl) in the cultivars ‘Arbosana’, ‘Arbequina’, ‘Chétoui’ and ‘Chemlali’ after 5 months of cultivation [144,159] and in ‘Ntopia’ after 6 months [153]. Conversely, no decrease in Fv/Fm has been detected in several cultivars grown for 6 months with high NaCl [157]. The decrease in Fv/Fm under salt stress indicates photoinhibitory damage, likely due to a direct effect of salt ions on leaf tissue, particularly on photosystem II [167,168]. Then, slight decreases in the Fv/Fm ratio affirm that PSII is relatively resilient to NaCl stress and that leaves possess effective photoprotection mechanisms to cope with salinity-induced stress [144,169].

Salt stress also affects olive trees by altering the photosynthetic pigment content [58,90,117,144]. A decrease in leaf chlorophyll content due to salt stress has been documented in different olive cultivars [57,58,90,91,144,154,170]. This decrease in chlorophyll content is a typical response associated with increased oxidative stress [171,172,173,174,175]. In fact, studies with different olive cultivars have shown a decrease in chlorophyll content and photosynthetic rate under salt stress correlates with increased catalytic activity of catalase and glutathione reductase in leaves [91,173,176,177]. 

In the ‘Chétoui’ and ‘Chemlali’ cultivars, increased carotenoid content and carotenoid/chlorophyll ratio were observed under salinity, which can be considered a protective response to prevent the photosynthetic apparatus from photooxidation [117,144]. Similar results were observed in ‘Picual’ plants pretreated with indole-3-acetic acid, salicylic acid, or quinetin and grown with 100 and 200 mM NaCl [58]. Carotenoids can act by quenching chlorophyll fluorescence directly by singlet energy transfer from chlorophyll to carotenoids or indirectly mediated by trans-thylakoid membrane-mediated and ΔpH [178,179,180]. In the ‘Arbequina’, ‘Koroneiki’, and ‘Chemlali’ cultivars, increased thermal energy dissipation and non-photochemical quenching associated with salinity were linked to an increase in the lutein/chlorophyll ratio [144].

### 2.3. Water Relations

The photosynthetic response, adjusted by stomatal conductance, leads to changes in water use efficiency (WUE) in plants exposed to osmotic stress [92,94,117]. The effect of salinity on WUE varies with the intensity and duration of the stress [94,153]. In saline environments, differences in WUE between cultivars have been observed [92,94,153]. Under salt stress, an increase in foliar WUE may be an indicator of salinity tolerance [94,181,182,183]. WUE increases when a decrease in stomatal conductance is greater than the decrease in photosynthesis. This may be a consequence of an increase in osmotic adjustment and hydraulic resistance and a decrease in photosynthesis due to stomatal limitation rather than a decrease in photosynthetic capacity [109,184]. In addition, the closure of stomata under salt stress reduces water loss by transpiration [94,109,114,117,155].

Leaf water potential decreased in different olive cultivars grown at different NaCl levels [93]. Olive is a drought-tolerant species and can, therefore, withstand quite low leaf water potentials without losing its turgor [183,185]. Olive can continue to transpire and photosynthesize at water potentials of −6 and −8 MPa [184,186] and can rehydrate in a short time after having lost 40% of the water in the tissues [187]. This is important for salt stress tolerance, as the first phase of salt stress is osmotic stress associated with cellular dehydration due to decreased water uptake and WUE [86,117]. Therios and Misopolinos [130] reported a decrease in water uptake in salinized olive trees, mainly caused by the decrease in osmosis in the external solution. Therefore, a reduction in relative leaf water content (RWC) by salinity has been described in different olive cultivars [91,92,118,154,155,188]. Differences in RWC have been described as a function of cultivar, NaCl level, and duration of salt stress [91,92]. Higher values of growth regulators and leaf water status under salt stress are related to a higher salt ion exclusion capacity of the shoot [91,92]. Furthermore, differences in a decrease in RWC of old and young leaves of the cultivar ‘Chondrolia Chalkidikis’ have been reported, where a decrease in RWC was about six times higher in old leaves than in young leaves [155].

More detailed ecophysiological studies are needed to understand how different olive cultivars respond to salt stress in various environments (i.e., different soil types and climates). In fact, the effectiveness of physiological adaptations requires in-depth research in a large series of environmental contexts. The analysis of structural changes at cellular and tissue levels is also critical to an overall understanding of these mechanisms. In parallel, the integration of physiological data with genomic analyses will be key to improving our scientific understanding of the olive tree’s adaptation to salt stress. This approach will enable us to identify cultivars that are naturally more tolerant, as well as guide the development of cultivation practices aimed at mitigating the negative effects of salt stress.

## 3. Biochemical Mechanisms in Olive for Adaptation to Salt Stress

Olive trees, renowned for their resilience in diverse environments, particularly saline stress, use not only physiological and morphological strategies but also biochemical mechanisms to thrive and persist in severe conditions.

### 3.1. Mineral Nutrients

Several studies have been carried out on the relationship between salinity and mineral nutrition in olive trees and have found it to be highly complex. Loupassaki et al. [189] studied the impact of salt stress on the concentrations of nitrogen (N), phosphorus (P), potassium (K^+^), calcium (Ca^2+^), magnesium (Mg^2+^), and sodium (Na^+^) in the leaves, shoots, and roots of six Greek olive cultivars (‘Koroneiki’, ‘Mastoidis’, ‘Kalamon’, ‘Amphissis’, ‘Kothreiki’, and ‘Megaritiki’). They observed a significant increase in Na^+^ levels in the various tissues of all cultivars, particularly in the roots, while K^+^ concentrations dropped in all organs. Similarly, Palm et al. [99] discovered that salinity induced a significant accumulation of Na^+^ in all tissues of olive cultivars ‘Frantoio’, ‘Leccino’, ‘Lecciana’, and ‘Oliana’, with the highest concentrations in the roots, while the K^+^ content decreased with salt level. They also detected a correlation between leaf Na^+^ concentration and a reduction in photosynthetic parameters. Bader et al. [97] investigated the salinity effect on three cultivars (‘Picholine’, ‘Meski’, and ‘Ascolana’) and revealed that the chloride concentration increased in roots with salinity but showed divergent trends in stems and leaves among cultivars. The potassium concentration decreased in all plant parts except for the cultivar ‘Picholine’, for which a notable increase in leaves was recorded, leading to significant decreases in the K^+^/Na^+^ and Ca^2+^/Na^+^ ratios under salinity treatment. In a recent study carried out on the ‘Leccino’ cultivar by Sodini et al. [114], salt treatment resulted in an accumulation of Na^+^ in the stems, while its concentration remained constant in the leaves. The same treatment induced a variation in Ca^2+^ levels in roots, while K^+^ and Mg^2+^ remained unchanged. They also noted a significant linear relationship between Na^+^ and Ca^2+^ in roots, indicating a potential interaction. Different patterns were also noted; for instance, the ‘Leccino’ cultivar accumulated Na^+^ in roots under saline conditions [111,113]. Lower concentrations of Ca^2+^, K^+^, and Mg^2+^ induced by salt stress have been observed by many authors [188,189,190].

Potassium deficiency in salinized plants is associated with an increase in Na^+^ accumulation, indicating the presence of ion competition effects [191]. The reduction in K^+^ concentration in olive roots, resulting in a low K^+^/Na^+^ ratio, may contribute to achieving ionic balance after the high uptake of sodium ions [192]. Polyphenols play a key role in reducing osmotic stress by improving the K^+^/Na^+^ ratio and water absorption, thus reducing oxidative stress in olive leaves under saline conditions [193,194,195]. Other studies have reported evidence of the influence of salinity–nitrogen interactions on plant growth and metabolism, proposing that N fertilization could mitigate the harmful effects of salinity; however, the precise mechanisms of this interaction, including nitrate reductase (NR) activity, require further investigation [157,196].

Cultivated plants, mainly adapted to low salinity, have difficulties efficiently absorbing and using nutrients in saline conditions because of the high concentrations of Na^+^ and Cl^−^ [197]. In fact, such conditions cause nutrient imbalances in plants as a result of various mechanisms, notably reduced availability, competitive absorption, and physiological inactivation of key nutrients such as potassium [198]. Olive cultivars display reduced growth and leaf damage when foliar Na^+^ levels exceed 0.4% of dry matter [134,149]. Effective mechanisms to mitigate salinity impacts in olives consist mainly of limiting the uptake and transport of sodium and chloride ions from the roots to the aerial parts, especially evident in salt-tolerant cultivars [17,89]. The ability to exclude sodium at low to moderate salinity levels helps to regulate ion concentration in xylem sap, thus preventing the toxic accumulation of ions in aerial parts; however, high salinity can lead to Na^+^ transport and accumulation in aerial parts, causing toxic symptoms [17,134]. Salt tolerance in olive cultivars involves efficient mechanisms for excluding and retaining Na^+^ and Cl^−^ ions in the roots, where calcium plays a crucial role in preserving the integrity of the plasma membrane; on the other hand, reduced Ca^2+^ uptake under salt stress is linked to reduced transpiration rates rather than direct competition with Na^+^ [89,135]. Tabatabaei [157] stated that nutrient availability and absorption in saline environments are dependent, in turn, on soil composition, solute concentration, pH, accompanying elements, and environmental factors such as aeration, temperature, and stress.

### 3.2. Osmotic Adjustment

#### 3.2.1. Proline Accumulation

Osmotic adjustment is an additional adaptive mechanism for olive trees in response to severe conditions. It involves organic compounds such as amino acids or soluble carbohydrates [199,200,201]. The process combines both active synthesis and accumulation of osmolytes in cells [202]. Proline, an essential amino acid, is involved in many different physiological processes in plants, particularly olive trees, and significantly influences their ability to tolerate salt exposure. Many studies have investigated the response of olive trees to saline conditions, revealing mainly the accumulation of proline as a result of its role in osmoregulation, although its exact function is not yet fully understood [92,119,203].

Recently, Trabelsi et al. [203] reported that full irrigation of a 26-year-old olive orchard (‘Chemlali’) with saline water (EC = 7.5 dS/m) increased the proline content in various organs compared with another fully irrigated with tap water (EC = 2.46 dS/m). 

This corroborates numerous previous findings; Ben Rouina et al. [119] observed significant proline accumulation under salt stress, with higher levels in leaves compared with roots, peaking during the summer season coinciding with low values of relative water content and water potential. The proline concentrations in olive leaves (‘Sigoise’) were found to be positively correlated with soil salinity, suggesting a potential involvement of proline in the osmotic regulation of cytoplasmic pH or N storage for post-stress periods [204]. Ayaz et al. [92] published a significant increase in proline levels in the leaves of three Turkish olive cultivars (‘Gemlik’, ‘Kilis’, and ‘Ayvalık’) subjected to different salt stress treatments. The proline content in olive leaves (‘Gemlik’) exhibited a significant increase with worsening salt stress severity, followed by a decline, which indicated that olive trees use proline synthesis as a tool for mitigating salinity-induced osmotic stress [205]. All of this reinforces the idea that proline accumulation correlates with the efficiency of salinity tolerance mechanisms in olive trees, suggesting that the time and magnitude of synthesis are probably linked to the level of salinity tolerance and biochemical strategies of cultivars [206].

On the contrary, in another investigation conducted by Regni et al. [91] on the salinity tolerance of the Croatian wild olive genotype and two well-known cultivars ‘Leccino’ and ‘Koroneiki’, the proline levels appeared unchanged or even decreased compared with the control. These authors supposed, in this case, that osmotic adjustment in olive leaves under hyperosmotic conditions could be achieved by the accumulation of K^+^ and other organic solutes rather than by the accumulation of proline. In a similar vein, Ben Abdallah et al. [117] recorded an 18% reduction in proline content in the leaves of salt-stressed plants (‘Chétoui’) irrigated every other day with 200 mM NaCl in 100% Hoagland’s solution, as compared with control conditions. The proline concentration was measured as a potential marker of cellular hydro–saline imbalance in leaves and showed a decrease during salt stress applied to ‘Royal’ and ‘Arbequina’ olive cultivars [207].

Despite its proven involvement in stress response, the precise function of proline is still widely debated but little clarified [116,160,208,209]. Various possible reasons have been suggested to explain the accumulation of proline in stressed plants. Ramanjulu and Sudhakar [210] attribute this accumulation to plant adaptation to salinity, while other studies propose that proline accumulation may result from salt stress-induced damage [211] or serve as an indicator of salt sensitivity [212], calling into question its role as a marker of plant salt tolerance.

Proline could protect the photosynthetic activity of salt-stressed olive trees by regulating hydration and osmotic adjustment, thus promoting growth even under stressful conditions [119]. Salt-stressed olive trees displayed an increasing proline concentration in their cytoplasm in order to enhance water absorption by tissues during active growth and to maintain ionic balance in vacuoles via osmotic adjustment effects [211]. Proline in the olive tree acts as a protective osmolyte in the face of environmental stresses, particularly saline conditions, both by reducing toxicity and by promoting osmotic regulation [213,214,215].

Proline can serve as a source of nitrogen in cells under stress conditions, where its accumulation could be utilized as a form of stored N, considering that proline contributed 12.16% of leaf nitrogen [216,217]. In situations of nitrogen limitation, proline functions as an alternative metabolic substrate under conditions of stress, therefore helping the maintenance of cellular energy and the balance between NADP^+^ and NADPH. This multifaceted role may be extended to its contribution to many pathways, including the tricarboxylic acid cycle and glutathione biosynthesis, which further emphasizes its dynamic nature as an organic reserve capable of supporting plant growth and meeting stress [218,219]. Ben Ahmed et al. [160,220] documented that proline supply improved photosynthetic activity and antioxidant defense enzyme activities in stressed plants, and its application, both in the presence and absence of salinity, significantly influenced salt ion distribution in the leaves and roots of ‘Chemlali’ olive trees.

#### 3.2.2. Sugar Accumulation

The accumulation of osmolytes, notably soluble sugars, represents a key survival mechanism for plants under salt stress, allowing them to prevent dehydration and protect cells from oxidative damage [221,222]. This adaptation varies across species and depends on the dose and duration of stress exposure [85].

Trabelsi et al. [203] recorded a significant increase in soluble sugars in olive leaf and root tissues during salt and water stress, mainly synthesized in the leaves. Furthermore, these same authors, in an earlier study, detailed a potential osmo-protective role and stress tolerance for these accumulated soluble sugars, as it could also be linked to reduced vegetative growth shown during these stress periods [223]. Salt stress significantly induced a 51% increase in total soluble sugars in the leaves of young olive seedlings (‘Chetoui’) transplanted into 10 L pots [117]. Meanwhile, Tester and Davemport [115] stated that salt accumulation in the vacuole stimulates the synthesis of organic solutes, thus balancing the osmotic potential of the cytoplasm with that of the vacuole. The increase in soluble sugars in olive leaves and roots, associated with a reduction in starch, has been observed concurrently with the counteraction of their biosynthetic enzymes, offering a crucial mechanism for plant tolerance to various abiotic stresses, including salinity [224,225].

Olive leaves contain a wide variety of carbohydrates, including glucose, mannitol, fructose, arabinose, myo-inositol, xylitol, xylose, galactinol, galactose, sucrose, raffinose, stachyose, and many others. Among them, glucose and mannitol are the most abundant [226,227]. Moula et al. [73] found, in their investigation of the quantification of soluble sugars in young olive plants (‘Chemlali’ and ‘Koroneiki’) subjected to salt stress, a significant increase in the concentration of mannitol, which was the most abundant sugar, while glucose and fructose showed no variation under this stress. This confirms the proposal by Tattini et al. [228] that mannitol actively contributes to the osmotic adaptation process in olive trees. According to the same authors, the rise in mannitol concentration is not simply a result of salinity but rather functions as a responsive mechanism, while glucose is the main sugar involved in metabolic functions and storage in olive leaves. Gucci and Tottini [88] noticed an accumulation of soluble carbohydrates, especially mannitol, in salt-stressed ‘Frantoio’ and ‘Leccino’ olive cultivars, indicating their contribution to osmotic adjustment.

Ayaz et al. [92] assessed mannitol levels and mannitol dehydrogenase (MDH) activity in three olive cultivars (‘Gemlik’, ‘Ayvalık’, and ‘Kilis’) under varying degrees of salt stress to examine their contribution to osmotic protection. Their results showed a significant increase in mannitol content, together with changes in mannitol dehydrogenase activities in all cultivars as salt stress was raised, clearly revealing a correlation between mannitol levels and enzymatic activities. Similarly, Conde et al. [229] reported that olive trees (‘Galega Vulgar’) subjected to salt and drought stress coordinated mannitol levels and mannitol dehydrogenase activity in source tissues. Salinity greatly elevates mannitol and glucose concentrations in olive leaves, while other soluble carbohydrates remain unaffected [228]. Mannitol accumulation occurs earlier than glucose [93]. 14C-labeling pulsechase experiments supported a preferential allocation of carbon to mannitol synthesis in salt-stressed olive leaves, indicating a central role for mannitol in improving salt tolerance [88].

### 3.3. Cell Wall Modification

Lignin is a biopolymer that has been identified in the xylem and schlerenchyma cell walls of vascular plants, particularly olive [230]. Its main functions are to provide rigidity to cell walls and ensure the impermeability of the tracheary elements, which facilitate the transport of water and solutes via the vascular system [231]. Many authors have shown evidence of lignin accumulation during saline stress in several species [232,233,234]. Lignification of vascular constituents reduces water permeability in the apoplasm [235]. Sánchez-Aguayo et al. [234] highlighted a decisive role of lignin accumulation in the process of plant adaptation to salt stress.

Concerning olive trees, Ben Abdallah et al. [117] observed a notable increment in leaf lignin content of the ‘Chétoui’ cultivar under water and salt stress conditions, compared with the control. Sofo et al. [221] asserted that the increase in lignification during stress conditions corresponds to a tolerance strategy adopted by olive plants to strengthen cell walls mechanically. They assume that this reinforcement can reduce water loss and cell dehydration by displacing the space occupied by mesophyll water, which, unlike lignified tissue, exchanges readily with the transpiration stream.

In salt stress conditions, olive leaves exhibit overexpression of two protein isoforms (Fra e 12.10) in olive leaves, which show strong sequence similarity to the main allergens of *Fraxinus excelsior* (European ash) [236]. Ben Abdallah et al. [117] suppose that this overexpression is related to lignin/lignan metabolism and suggest that lignification may be a tolerance strategy adopted in olive trees to strengthen cell walls and reduce water loss.

### 3.4. Antioxidant Defense Activity

**Figure 5:**
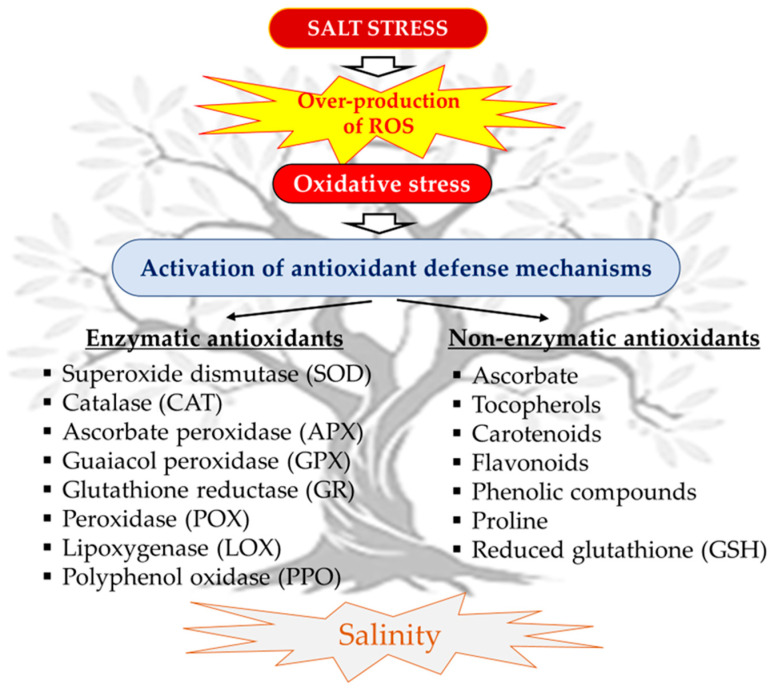
Outline of the antioxidant defense system of the olive tree under salt stress.

Salt stress has a significant impact on plants by inducing oxidative stress through the excessive production of reactive oxygen species (ROS), which can damage vital cellular constituents. The olive tree has various mechanisms to prevent these damaging effects, thanks to its antioxidant defense system (Figure 5). 

This system consists of enzymes such as superoxide dismutase (SOD), catalase (CAT), ascorbate peroxidase (APX), and guaiacol peroxidase (GPX) [221], while the non-enzymatic system includes substances with antioxidant properties such as ascorbate, tocopherol, carotenoids and various phenolic compounds [230].

Ayaz et al. [92] reported that salt stress caused increased levels of reactive oxygen species, which require an active antioxidant defense system for tolerance, thus suggesting that antioxidant enzyme activity could serve as a marker of salinity tolerance in olive cultivars. They analyzed the activities of SOD, GPX, glutathione reductase (GR), peroxidase (POX), and CAT in response to higher salinity levels and observed a notable increase in three olive cultivars ‘Gemlik’, ‘Ayvalık’, and ‘Kilis’. Regni et al. [91] documented a systemic increase in GSH and CAT activities in the leaves of salt-treated olive plants (‘Fadak 86’, ‘Royal de Cazorla’, ‘Koroneiki’, and ‘Arbequina’). Similarly, Sofo et al. [221] found higher activities of antioxidant enzymes, namely APX and CAT, as well as SOD and peroxidase (POX), lipoxygenase (LOX) activity, and malonaldehyde (MDA) concentrations, indicating membrane lipid oxidation, which is linked to photosynthetic apparatus damage. The same authors also detected a lower polyphenol oxidase (PPO) activity to protect against phenol oxidation. These enzymes are widely documented to be activated in response to abiotic oxidative stress by plants [152,208,237,238,239]. They play a central role in protecting chloroplasts from oxidative damage [163]. Plants have been shown tolerance to saline conditions and succeed in thriving even under these severe environments thanks to a significant increase in the production of antioxidant enzymes [206,240,241].

Salt-tolerant species induce the production of antioxidant enzymes, thus improving their ability to eliminate reactive oxygen species. In contrast, salt-sensitive species exhibit a reduction in antioxidant activity [242]. This decline in enzymatic activity caused an accumulation of hydrogen peroxide and malondialdehyde. Ertani et al. [243] explained the accumulation of hydrogen peroxide as the result of reduced APX when exposed to salt stress. Del Buono et al. [154] observed that salt exposure to olive plants (‘Arbequina’) caused significant reductions in the activities of antioxidant enzymes, especially ascorbate peroxidase (APX) to a large extent, and others such as SOD, GPX, and CAT. In another similar study, Bano et al. [244] found that salinity strongly reduced SOD, CAT, and peroxidase activities, as well as this effect was cultivar-dependent. 

The total antioxidant capacity of olive leaves (‘Changlot Real’, ‘Picual’, and ‘Arbequina’) was significantly reduced under salinity stress [245]. The antioxidant activity, in particular that of SOD, increased in response to salt stress, thus reducing oxidative stress in olive trees, notably via non-enzymatic antioxidants such as polyphenols [246]. Iwaniuk et al. [247] reported the relevance of SOD, in conjunction with other antioxidants (CAT and NADH-dependent peroxidase [POD]), in the plant defense system against biotic stress as well as by managing ROS levels and reducing oxidative damage. Total phenols and phenolic compounds are involved in the antioxidant mechanisms developed by the olive tree in response to oxidative stress induced by salt stress [206,220].

In some olive cultivars (‘Gemlik’, ‘Nizip Yaglık’, and ‘Kilis Yaglık’), the salt treatment showed no significant impact on polyphenol and protein content, but it reduced total flavonoid content, indicating a consistent response to salt-induced stress [248]. Phenolic compounds play a crucial role in combating abiotic and biotic stress drivers, as both their biosynthesis and accumulation are activated by these factors, although some studies indicate a decrease in their content as stressors increase [249,250]. Flavonoids have been reported to improve the ability of trees to tolerate stress through their influence on physiological performance, leading to reduced lipid peroxidation [251]. Demiral et al. [205] noted a change in the leaf antioxidant activity of the ‘Gemlik’ olive cultivar during salt stress, linked to proline and total phenol levels. Proline enhances photosynthetic activity and antioxidant defense enzymes more effectively, as well as acting on the distribution of salt ions in olive trees [220].

### 3.5. Lipid Peroxidation

Salt stress can also alter the structural properties and integrity of membranes, leading to their deterioration. Many researchers have used lipid peroxidation analysis to assess the damage inflicted on olive trees by salt stress.

Ben Abdallah et al. [117] conducted a study on the effect of salt stress on oxidative damage in Chétoui variety leaves and revealed a 50% rise in lipid peroxidation, as measured using (malondialdehyde) MDA content, compared with the control. They speculated that salt-stressed olive trees might accumulate both reactive oxygen species and electrolytes, leading to heightened oxidative damage. Ayaz et al. [92] have determined MDA levels in the leaves of olive cultivars ‘Gemlik’, ‘Kilis’, and ‘Ayvalık’, and also revealed some spectacular increases in MDA content, with differences observed among varieties. Several other research reports have cited similar trends of significant rises in MDA under salt stress [152,154,204].

Recently, Lima-Cabello et al. [245] have analyzed lipid peroxidation as an indicator of salinity resistance by measuring the combined levels of malondialdehyde (MDA) and 4-hydroxynonenal (HNE) [MDA + HNE] in commercial olive trees subjected to saline treatments. The results showed higher levels of MDA and HNE in the ‘Arbequina’, ‘Cobrançosa’, ‘Pico-Limón’, and ‘Cornezuelo’ cultivars compared with controls, while no significant difference was observed for the ‘Changlot Real’ and ‘Picual’ cultivars, renowned for their resistance to salinity. Similarly, Azariadis et al. [252] found that MDA levels increased significantly in salt-sensitive varieties (‘Koroneiki’ and ‘Arvanitoli’) but not in certain tolerant varieties (‘Lefkolia’ and ‘Gaidourelia’) when all were exposed to saline conditions. Meanwhile, in another study, MDA levels in two olive cultivars (‘Canino’ and ‘Sirole’) under different salt concentrations remained unchanged [253]. Non-significant MDA variations were found in the roots of olive cultivar ‘Leccino’ plants under salt stress [114].

### 3.6. Adaptations at the Protein Level

Different defense mechanisms are triggered when plants are subjected to salt stress, mobilizing a complex set of proteins to mitigate the adverse effects of salinity on growth and development. For olive trees, the crucial role of proteins and their involvement in salt stress tolerance are the subject of intense investigations.

Bashir et al. [253] reported a close relationship between salt stress and protein concentration in ‘Canino’ and ‘Sirole’ olive cultivars. Demir and Cetinkaya [248] stated a slight change in protein content when olive cultivars ‘Gemlik’, ‘Kilis Yaglık’, and ‘Nizip Yaglık’ were exposed to high salinity levels. Valderrama et al. [254] conducted a research study on olive response to salt stress and found a 30–50% increase in protein content. Similarly, Parida et al. [255] affirmed that salinity rise can induce changes in free amino acid levels and reduce total protein content, thereby boosting the activity of acid and alkaline proteases, thus offering resistance to stress conditions. The accumulation of these proteins constitutes a source of nitrogen for osmotic adjustment [256]. Salt stress-induced proteins can be classified into two main groups: salt-stress proteins, which accumulate specifically in response to salt stress, and stress-related proteins, which accumulate in response to various abiotic stresses [257]. 

Arabinogalactan proteins (AGPs) are key glycoproteins in plant cell walls, with a significant role in growth, development, and responses to environmental stresses [258]. A related claim was reported by Ouyang et al. [259], who consider wall-associated kinases and AGPs to be potential candidates for detecting stress-induced changes in plant physiology. The same authors noted that transcriptional analyses have indicated a repression of cell wall-related gene expression, including AGPs and xyloglucan, as a result of salt stress. Similarly, Azariadis et al. [252] reported that immunodetection revealed a weak AGP signal in the ‘Koroneiki’ cultivar, which was associated with irregular cells and intercellular spaces, resulting in the formation of aerenchyma after 45 days of salt treatment. Hydroxyproline-rich cell wall glycoproteins (HRGP), such as arabinogalactans proteins (AGP), are involved in salinity stress [260,261].

Ben Abdallah et al. [117] achieved reproducible 2-DE proteomic maps of ‘Chetoui’ olive leaves for the first time in an original investigation. The analysis of these maps identified 26 differentially expressed protein spots, with the majority corresponding to proteins involved in photosynthesis. Other identified proteins were associated with nitrogen metabolism, protein storage, energy, and other functional categories. The profiles of the identified proteins in control and salt samples are provided, revealing significant differences in expression patterns. Under salt stress conditions, several photosynthesis-related proteins showed reduced expression levels compared with the control. These included ribulose-1,5-bisphosphate carboxylase/oxygenase (RuBisCO) large subunit isoforms, oxygen-evolving enhancer protein 1 (OEE1) isoforms, oxygen-evolving enhancer protein 2 (OEE2) isoforms, ferredoxin-NADP reductase (FNR), BSP, glutamine synthetase cytosolic isozymes (GSc), salicylic acid-binding protein (SABP), and carbonic anhydrase isozyme (CA), while others such as ATP synthase subunit β, GS nodule isoenzyme (GSn), and Fra e 12.10 allergen isoforms were over-expressed. The down-regulation of RuBisCO isoforms and some other proteins in saline stress conditions indicates a limitation of photosynthetic efficiency due to factors linked to low intercellular CO_2_ and reduced electron transport activity [262,263]. Moreover, the down-regulation of enzymes involved in CO_2_ maintenance and salicylic acid signaling, such as CA and SABP, respectively, suggests possible mechanisms for reduced stress tolerance [264,265,266,267]. In addition, the overexpression of specific proteins, such as Fra e 12.10, may reflect an adaptive response, including lignification, to mitigate stress effects [268,269,270]. Alterations in cytoplasmic proteins affect cell viscosity under saline conditions [271].

Clarifying the complex biochemical mechanisms governing the olive tree’s adaptation to salt stress continues to be a major frontier in plant biology and agronomy. Integrative and multidisciplinary research is required to fill these knowledge gaps in order to ensure the future of this emblematic Mediterranean crop in the face of environmental challenges. Gene regulation, signaling pathways, and epigenetic modifications are still relatively unclear aspects that can be addressed through extensive explorations based on a combination of omics technologies, functional genomics, advanced imaging, and field studies.

## 4. Molecular Mechanisms in Olive for Adaptation to Salt Stress

Plant adaptation or tolerance to salt stress involves a complex process of physiological and biochemical traits and metabolic pathways controlled by a network of genes [272,273,274]. This process begins with detecting stress signals, followed by signal transduction that activates stress-responsive genes, ultimately leading to metabolic adjustments able to counter salinity-induced osmotic stress and other various stressors [275,276].

From the recent literature on olive tree responses to salinity, we can note a serious gap concerning the molecular network that governs its adaptation to such severe conditions compared with the physiological, biochemical, and metabolic mechanisms that have been widely investigated. The lack of molecular research into olive resistance to salt stress has recently been filled by some studies seeking both to identify the genes induced by stress and to analyze their functions.

Bazakos et al. [131] conducted a comparative transcriptomic analysis of two olive cultivars under salt stress conditions, which is widely acknowledged as a seminal study in understanding the molecular response of olive trees to salt stress. They found that in the cultivar ‘Kalamon’, 159 transcripts consistently showed up-regulation during stress, with 50 others exhibiting delayed up-regulation, but all 209 were down-regulated post-stress. In contrast, the cultivar ‘Chondrolia Chalkidikis’ displayed limited transcriptional activation (20 transcripts), leading the authors to suggest that this reduced transcriptional response might be partly responsible for the sensitivity of the cultivar ‘Chondrolia Chalkidikis’ compared with the cultivar ‘Kalamon’ in terms of gene expression. Seven transcripts in the cultivar ‘Kalamon’ were recognized by these authors as salt-specific, namely putative small glutamine-rich tetratricopeptide repeat-containing protein, 40S ribosomal protein, NAD^+^ ADP-ribosyltransferase, annexin A4, xyloglucan endotransglycosylase, UDP-galactose epimerase, and stress-induced protein. JEREF and bZIP transcription factors have been identified as key regulators of the response of olive trees to salt stress, with differences in expression between the two cultivars indicating their importance in salt tolerance. Zhang et al. [277] reported the involvement of JERF in the response to abiotic stress, including salinity, by regulating the expression of stress-responsive and ABA biosynthesis-related genes. Similarly, Yoshida et al. [278] showed that AREB1, a group A bZIP, is crucial in ABA signaling in response to drought stress.

In further research, Bazakos et al. [279] used next-generation sequencing to explore the molecular basis of the salt stress response in the olive cultivar ‘Kalamon’, focusing on leaves and roots. Transcriptomic analysis disclosed 24 genes differentially expressed in roots (9 down-regulated and 15 up-regulated) and 70 in leaves (14 down- and 56 up-regulated), giving tissue-specific responses to salt stress. The differentially expressed transcripts are consistent with findings in chickpeas and maize, suggesting common stress response pathways to plant species [280,281]. In addition, the authors stated that BlastX analysis identified fourteen transcripts associated with cell wall hydrolases (eight down-regulated in roots and only one in leaves), which could have an impact on wall elasticity under salt stress. A reduction in hydrolase activity may decrease wall elasticity, making plants more rigid and tolerant to salt stress [280]. In addition, Bazakos et al. [279] noted that 19 clusters encode cytochrome P450s, showing significant differential expression in leaves and roots. This gene family is involved in lignin biosynthesis and is known for its role in the response to abiotic stress [282].

Rossi et al. [113] assessed the expression levels of genes implicated in the phenylpropanoid metabolic pathway, compounds known to be specialized in responses to environmental stimuli, in ‘Leccino’ (salt-sensitive) and ‘Frantoio’ (salt-tolerant) cultivar olive plants grown in a phytotron chamber and treated with NaCl. In this study, they highlighted the impact of salt stress on gene expression along phenolic pathways in olive trees, revealing a variable response depending on the organ, genotype, and specific enzyme involved. Phenylalanine-Ammonia-Lyase (PAL), the primary enzyme converting phenylalanine into cinnamic acid, is up-regulated in response to salinity, varying by organ and genotype. The expression of Cinnamate-4-Hydroxylase (C4H), involved in the conversion of cinnamic acid to p-coumaric acid, remained stable in all treatments at most organs, except for significant up-regulation in ‘Leccino’ roots under moderate salt stress. The enzyme 4-coumarate: CoA ligase (4CL), responsible for 4-coumarate activation, showed higher expression in ‘Leccino’ aerial organs, with up-regulation in leaves. Chalcone Synthase (CHS) and Chalcone Isomerase (CHI), key enzymes in flavonoid biosynthesis, exhibited low expression levels but were up-regulated in new leaves of both cultivars under NaCl stress. The same authors observed that expression of the Na^+^/H^+^ Exchanger (NHX) gene, a recognized molecular marker of salt stress in other plant species [283], was significantly increased in all ‘Leccino’ organs in response to salt treatment, while it remained insignificant in ‘Frantoio’, indicating a differential perception of salt stress between the cultivars.

Said [274] focused his investigation more specifically on the change in expression of the AtTPS1 gene, which codes for trehalose-6-phosphate synthase (TPS), in salt-stressed plants. This study was conducted on two olive cultivars, ‘Pecaul’ (salt-tolerant) and ‘Aggizi Shame’ (moderately salt-tolerant), which were treated with validamycin A and grown under saline conditions. The results revealed successful amplification of AtTPS1 in both cultivars, with an additional fragment at 210 bp observed, particularly in the salt-tolerant cultivar (‘Pecual’). Trehalose synthesis relies on the enzymes trehalose-6-phosphate synthase (TPS) and trehalose-6-phosphate phosphatase (TPP). The increase in cytoplasmic trehalose induced by validamycin A inhibits TPP activity, increasing trehalose-6-phosphate (T6P) levels [284]. Accordingly, the results of Said [274] suggest the involvement of olive TPS in salt stress response with validamycin A in both cultivars. AtTPS1 and AtTPP genes have previously been discovered in Arabidopsis thaliana, emphasizing their relevance to plant stress responses [285,286,287]. Penna [288] asserted the potential strategy of modulating trehalose levels, a key player in the stress response, either through inhibition of trehalase or enhancement of its biosynthesis.

Mousavi et al. [289] analyzed the differential regulation of candidate genes putatively for the salt stress response across four olive cultivars ‘(Koroneiki’, ‘Picual’, ‘Royal de Cazorla’, and ‘Fadak86’). They revealed that only OeNHX7, OeP5CS, OeRD19A, and OePetD exhibited up-regulation in tolerant cultivars (‘Royal’ and ‘Fadak86’), indicating their key role in the activation of a salt tolerance mechanism. Up-regulation of NHX, as observed with OeNHX7, was related to an enhanced accumulation of Na^+^ in the vacuole, hence contributing to mitigating salt stress [148,283]. P5CS gene expression has been shown to improve salt tolerance through proline accumulation, which regulates Na^+^ accumulation in leaves [290,291,292]. The cysteine protease genes RD21A and RD19A are involved in the programmed cell death pathway during stress [293]. The up-regulation of OeRD19A supports its implication in improving salt tolerance in olive trees [289]. The chloroplast gene PetD (CytB6), a component of the plastoquinone-plastocyanin reductase, plays a critical role in electron transport and ATP production, thus offering a defense against salinity-induced oxidative damage [289,294,295].

Recently, Sodini et al. [114] delved into the molecular mechanisms underlying the response of ‘Leccino’ olive tree (salt-sensitive) roots to NaCl stress, focusing on the expression of genes associated with proton pumps and Na^+^/H^+^ exchangers. They specifically examined genes such as PATPase, V-type ATPase sub E, SOS1, and NHX, designing primers based on the *Olea europaea* genome and genes from other tree species. They revealed that salt treatment resulted in a decrease in the expression of P-ATPase 1 and SOS1 after 24 h, followed by NHX and V-ATPase sub E after 48 h, and ultimately P-ATPase 8 within 7 days. Membrane H^+^-ATPase genes are typically under-expressed in response to salinity [296]. P-type H^+^-ATPase has housekeeping functions, aiding turgor pressure and cell wall extension [297]. SOS1 gene in roots functions in Na^+^ exclusion and loading into the xylem, potentially leading to sodium accumulation in leaves [115,150,298]. V-ATPase subunits and NHX genes are involved in the mechanisms of root Na^+^ storage, and their down-regulation aligns with the observed lack of sodium accumulation in the roots [299].

The genetic and molecular pathways that ensure salt tolerance in olive trees are not well understood. Future research should focus on detecting and deciphering specific genes and their regulatory networks involved in the response to salt stress. More advanced techniques could prove invaluable in uncovering these pathways. In addition, investigations into DNA methylation, changes in histones, and non-coding RNAs could provide new insights into the inherited properties and complexity of salt stress responses in olive trees.

## 5. Factors Affecting Salinity Tolerance

The olive tree is widely recognized for its resilience and ability to tolerate severe environments [117,300,301], including those with high levels of salinity, thanks to a series of adaptive mechanisms [119,121,244,246]. Nevertheless, the extent of its tolerance to saline stress is also conditioned by many other factors. Studies have revealed significant variation in the response of olive trees to stress depending on the cultivar, as some cultivars such as ‘Leccino’, ‘Arvanitoli’, and ‘Arbequina’ displayed salt sensitivity, while others such as ‘Frantoio’, ‘Lefkolia’, ‘Gaidourelia’, ‘Manzanillo’, and ‘Hojiblanca’ showed more resilience [113,252]. The specific genetic characteristics of each cultivar define its salt tolerance, thereby affecting ion absorption/exclusion regulation, osmolyte accumulation, resistance to oxidative stress, and the activation of metabolic pathways [131,274,288]. Furthermore, prior investigations have demonstrated that both young olive trees and organs are more susceptible to salt stress compared with their older counterparts [157,302,303]. The effects of salt stress on olive trees are not limited to cultivar tolerance. The physiological, biochemical, and molecular responses of the olive tree are in part dependent on the concentration and nature of salts and nutrients available in the soil and irrigation water. In fact, high salt concentrations exacerbate the damaging actions of salt stress on olive trees [99,253].

Several other environmental factors are critical in regulating the response of olive trees to saline conditions. Soil composition and pH, as well as solute and nutrient content, all influence ion exchange [157]. In addition, water availability has proven to be essential for the olive tree in managing salt in the soil; unbalanced conditions, particularly a water shortage, may exacerbate water stress and increase salt concentration [117], which further emphasizes the importance of finding the right balance to support the resistance of the olive tree to salt stress. High temperatures can also aggravate the effects of salt stress by accelerating transpiration [304]. Hence, we also hypothesize that cultivation and management practices, such as irrigation methods and fertilization, may influence the salinity tolerance of the olive tree.

## 6. Conclusions

Olive cultivation has experienced rapid expansion worldwide, although its primary production remains concentrated in the regions of the Mediterranean and the Middle East. However, these regions are facing major challenges associated with demographic pressure and climate change, significantly impacting water availability and quality. This situation is giving rise to the growing risk of soil and water salinization, particularly threatening agricultural production, including that of olives and olive oil.

The literature on the response of the olive tree (*Olea europaea*) to saline conditions has revealed multiple tolerance mechanisms to cope with these stressful environments. The olive tree may undergo structural changes in the root to favor ion exclusion from the shoot. It can remove senescent leaves where there is an accumulation of toxic ions and adjust its morphology to limit water loss caused by salt stress, in particular by reducing growth, especially of the aerial part, and modifying leaf anatomy. In addition, stomatal conductance is increased by salinity, leading to a decrease in transpiration and photosynthesis and an increase in water use efficiency. In fact, the decrease in photosynthesis is mainly due to stomatal closure since most studies revealed that PSII is not very susceptible to saline ions. Moreover, to resist and adapt to salinity, the olive tree relies on biochemical mechanisms that include the accumulation of osmolytes, such as sugars and amino acids, as well as the regulation of ionic flux to maintain osmotic and ionic balance. Additionally, research has unveiled the synthesis of specific metabolites and enzymes crucial for protecting plant cells against oxidative damage induced by salt stress. In contrast, molecular exploration of the response of olive trees to salt stress has lagged behind the other aspects; recently, considerable attention has been devoted to understanding the expression and functions of involved genes, especially ion transporters and signaling proteins that regulate olive responses to saline conditions.

Profiling genes contributing to physiological and biochemical processes related to salt tolerance in olive trees could enable the identification of specific markers. Understanding all these mechanisms is essential for the development of selection and breeding strategies to improve the ability of olive trees to grow in saline environments, thus ensuring their sustainability.

## Figures and Tables

**Table 1:** List of the most important cultivars of each continent [7].

Continents	Main Cultivars
Africa	Picholine, Marroqui, Hacuzia, Meslala, Menara, Arbosana, Arbequina, Koroneiki, Woira, Mission, Kalamata
America	Arbequina, Azapá, Criolla, Coratina, Barouni, Manzanilla, Ascolana, Mision, Arauco, Picus
Asia	Arjosi, Barmagui, Basica, Kikkam, Kasb, Souri, Nabal Baladi, Mehravia, Muhasan, Mastoidis
Europe	Arbequina, Picual, Hojiblanca, Koroneiki, Arbosana, Alentajana, Frantoio, Verdial, Picudo, Carbonella
Oceania	Hardy’s, Mammoth, Fs17, Dai21, Azapa, Picual, Hojiblanca, Manzanilla, Barnea, Frantoio, Koroneiki
World	Arbequina, Arbosana, Koroneiki, Picual, Frantoio, Leccino, Hojiblanca, Verdial, Kalamata, Picholine, Alentajana, Nabal Baladi

**Table 2:** Continental distribution of the area of saline soils (Mha). Prepared by the authors based on data from Mukhopadhyay et al. [19], Wike et al. [23], Ivushkin et al. [24], and Kramer et al. [25].

Continents	Area (Mha)	%	Main Countries
Africa	80.44	0.86	Algeria, Egypt, Ethiopia, Lybia, Mali, Mauritania, Tunisia, Western Sahara,
America	144.73	1.55	Argentina, Bolivia, Chile, Mexico, USA
Asia	316.50	3.40	China, India, Iran, Iraq, Mongolia, Oman, Saudi Arabia, Yemen,
Europe	30.70	0.33	Norway, Spain
Oceania	357.33	3.84	Australia
World	931.70	100	-

## Supplementary Materials

The following supporting information can be downloaded at: https://www.mdpi.com/article/10.3390/plants13152094/s1, Figure S1: Area harvested (Mha), production (t), destination of olives (Mha), and yield (t/ha) of olive grove cultivation in 2021. Figure S2: Area (Mha) of rainfed, and irrigated olive grove cultivation, and area (%) of low-density, intensive and super-intensive olive grove cultivation in 2021. Figure S3: Average table olive (× 10^3^ t) and olive oil production (× 10^3^ t) in the main producing countries from 2013 to 2016.
