# Supplementary material for: Tolerance Mechanisms of Olive Tree (Olea europaea) under Saline Conditions
Source: Plants (Basel). 2024 Jul 29;13(15):2094. doi: 10.3390/plants13152094 (PMC11314443; doi:10.3390/plants13152094)
Supplement: Supplementary file 1 [file plants-13-02094-s001.zip › plants-3042117-supplementary.pdf]

Supplementary Figures S1, S2 and S3

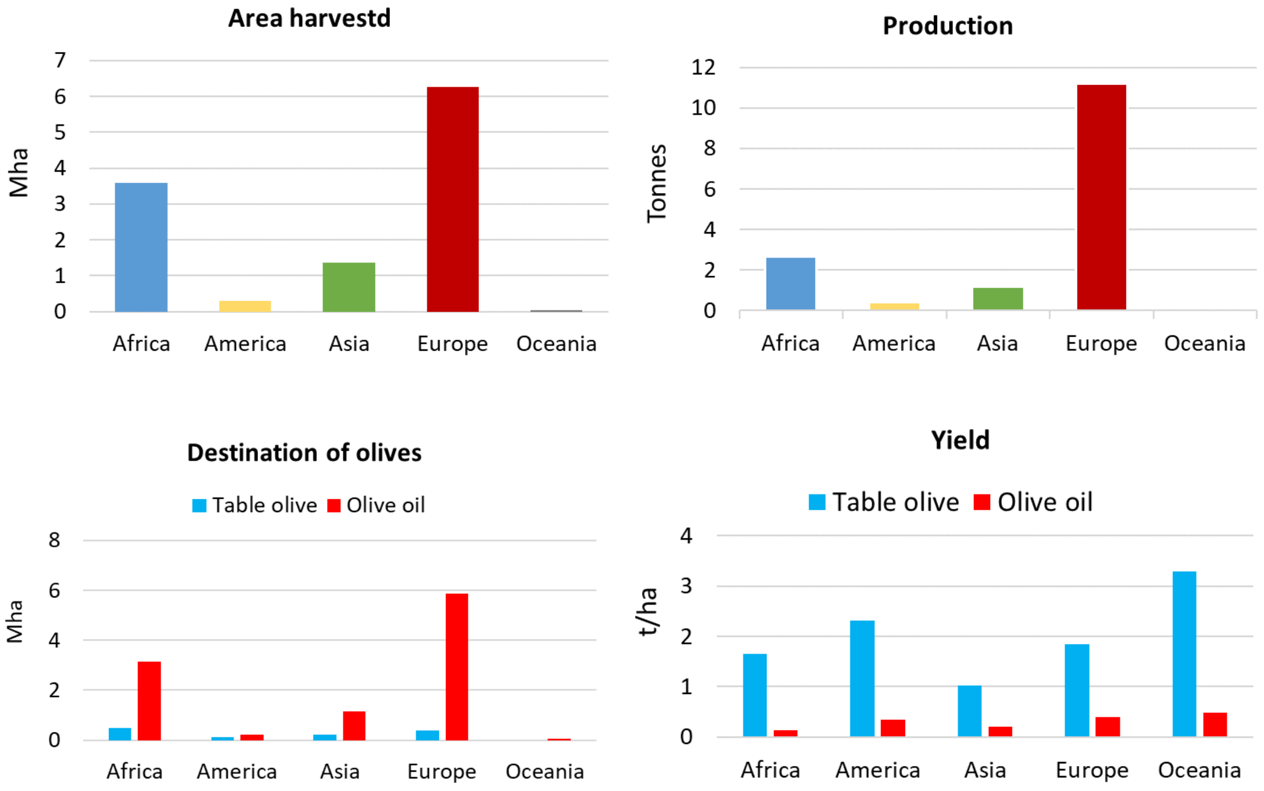

Figure S1. Area harvested (Mha), production (t), destination of olives (Mha), and yield (t/ha) of olive grove cultivation in 2021. Prepared by the authors based on data from the IOC [1].

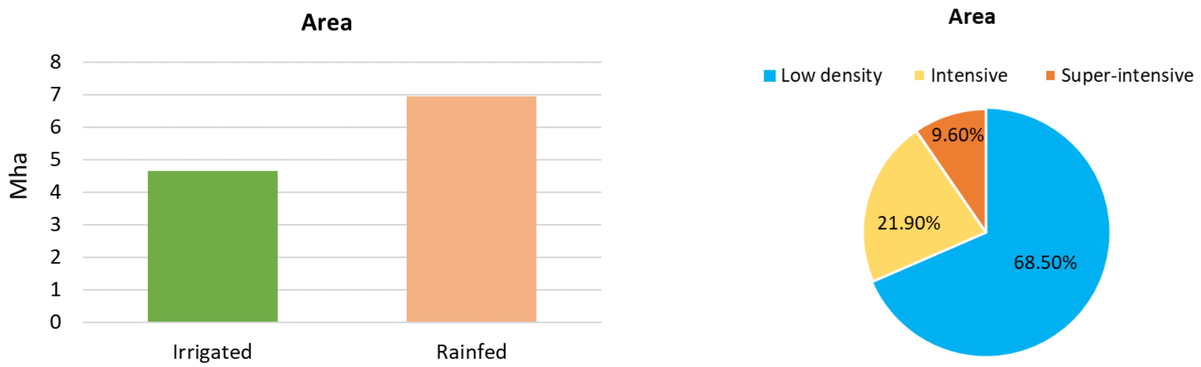

Figure S2. Area (Mha) of rainfed, and irrigated olive grove cultivation, and area (%) of low-density, intensive and super-intensive olive grove cultivation in 2021. Prepared by the authors based on data from the IOC [1].

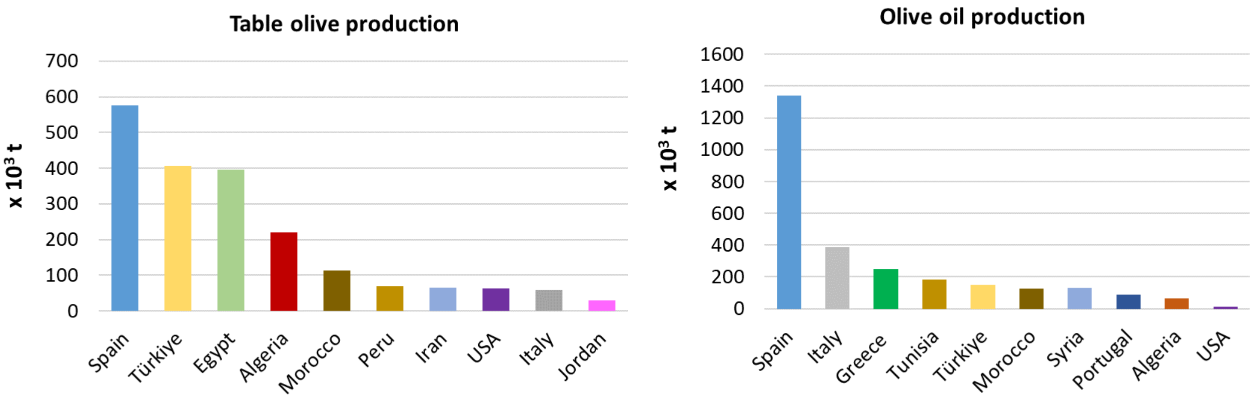

Figure S3. Average table olive (x 10^3 t) and olive oil production (x 10^3 t) in the main producing countries from 2013 to 2016. Prepared by author based on data from the IOC [2].

References

1. IOC. International Olive Council. 2022. Available online: https://www.internationaloliveoil.org (accesed on 11 April 2024).

2. IOC. International Olive Council. 2018. Available online: https://www.internationaloliveoil.org (accesed on 11 April 2024).
